# Dysregulation of Autophagy Contributes to Anal Carcinogenesis

**DOI:** 10.1371/journal.pone.0164273

**Published:** 2016-10-05

**Authors:** Evie H. Carchman, Kristina A. Matkowskyj, Louise Meske, Paul F. Lambert

**Affiliations:** 1 Department of Surgery, University of Wisconsin, Madison, WI, United States of America; 2 Department of Pathology and Laboratory, University of Wisconsin, Madison, WI, United States of America; 3 Department of Oncology, University of Wisconsin, Madison, WI, United States of America; Universidad Complutense de Madrid, SPAIN

## Abstract

**Introduction:**

Autophagy is an intracellular catabolic process that removes and recycles unnecessary/dysfunctional cellular components, contributing to cellular health and survival. Autophagy is a highly regulated cellular process that responds to several intracellular signals, many of which are deregulated by human papillomavirus (HPV) infection through the expression of HPV-encoded oncoproteins. This adaptive inhibitory response helps prevent viral clearance. A strong correlation remains between HPV infection and the development of squamous cell carcinoma (SCC) of the anus, particularly in HIV positive and other immunosuppressed patients. We hypothesize that autophagy is inhibited by HPV–encoded oncoproteins thereby promoting anal carcinogenesis (Fig 1).

**Materials and Methods:**

HPV16 transgenic mice (K14E6/E7) and non-transgenic mice (FVB/N), both of which do not spontaneously develop anal tumors, were treated topically with the chemical carcinogen, 7,12-Dimethylbenz[a]anthracene (DMBA), to induce anal cancer. The anuses at different time points of treatment (5, 10, 15 and 20 weeks) were analyzed using immunofluorescence (IF) for two key autophagy marker proteins (LC3β and p62) in addition to histological grading. The anuses from the K14E6/E7 mice were also analyzed for visual evidence of autophagic activity by electron microscopy (EM). To see if there was a correlation to humans, archival anal specimens were assessed histologically for grade of dysplasia and then analyzed for LC3β and p62 protein content. To more directly examine the effect of autophagic inhibition on anal carcinogenesis, nontransgenic mice that do not develop anal cancer with DMBA treatment were treated with a known pharmacologic inhibitor of autophagy, chloroquine, and examined for tumor development and analyzed by IF for autophagic proteins.

**Results:**

Histologically, we observed the progression of normal anoderm to invasive SCC with DMBA treatment in K14E6/E7 mice but not in nontransgenic, syngeneic FVB/N background control mice. With the development of low-grade dysplasia in the K14E6/E7 mice, there was an increase in both punctate LC3β and p62 expression while EM revealed increased autophagosomes without evidence of autophagolysosomes. These observations are consistent with autophagy being inhibited at a later stage in the autophagic process. In contrast, in high-grade dysplasia and SCC in the DMBA-treated K14E6/E7 mice, there were decreased levels of p62 with a continued increase in punctate LC3β expression by IF, while autophagolysosomes were seen on EM, consistent with the process of autophagy proceeded to completion. Similar findings, including histological grade dependent changes in LC3β and p62 expression, were noted with human samples upon analysis of IF. Finally, with pharmacologic inhibition of autophagy in DMBA-treated, nontrangenic FVB/N mice, there was a significant increase in anal cancer development similar to that observed in DMBA- treated K14E6/E7 mice.

**Conclusion:**

Autophagic dysregulation is noted early on in HPV-associated anal carcinogenesis (low-grade dysplasia), with normalization of the autophagic process arising in late stages of HPV-associated anal carcinogenesis (high-grade dysplasia and invasive carcinoma).

## Introduction

Squamous cell carcinoma of the anus is a rare gastrointestinal cancer whose incidence and mortality are increasing at a rate of 2.2% and 3.2% per year, respectively [1]. The majority of anal cancer cases are squamous cell carcinomas, and are associated with ‘high-risk’ human papilloma virus (HPV) infection of the anal mucosa. HPV infection of the anus has been identified as the major initiating factor in the development of anal carcinoma, with as many as 95% of biopsies testing positive for one or more genotypes of high-risk HPV [2]. HPV infection of epithelial cells is known to result in the production of several viral-associated oncoproteins such as E5, E6, and E7. E6 and E7 oncoproteins are universally expressed in *all* HPV-positive anal carcinomas. These oncoproteins modulate normal cellular pathways to enable infected cells to grow in an uncontrolled manner, disengage normal pathways such as programmed cell death, and prevent viral clearance. Each of these intracellular changes are adaptive to allow for viral survival and proliferation in the context of the innate and adaptive host immune responses. The intracellular changes initiated by the HPV oncoproteins allow for viral persistence, but also create an environment supportive of carcinogenesis. In isolation, these two oncoproteins are *insufficient* for carcinogenesis. However, their expression results in changes in intracellular processes that are important for monitoring cellular health and preventing the accumulation of genomic damage, thus contributing to carcinogenesis in an already primed intracellular environment [3].

One intracellular process that E6 and E7 are known to modulate, through actions on upstream regulators, is autophagy. It is also noted that in the majority of human anal cancer cases (60%) there are activating gene alterations in the upstream inhibitors of autophagy (PI3K/AKT/mTOR), which promote autophagic inhibition [4]. Autophagy is an evolutionarily well-conserved intracellular catabolic process wherein intracellular proteins and organelles undergo targeted lysosome-mediated degradation. Proteins and organelles undergoing autophagic degradation are isolated from the cytoplasm in a double membrane vesicle called an autophagosome. The autophagosome then fuses with a lysosome to form an autophagolysosome and the contents of the autophagosome are degraded. Autophagy is important in the maintenance of cellular health through the removal of dysfunctional cellular constituents. This process also maintains cellular energetic homeostasis through the creation of energy from recycling cellular waste products. When autophagy is perturbed there is an accumulation of dysfunctional organelles, such as mitochondria, which produce damaging reactive oxygen species, leading to genomic oxidative damage and release of pro-apoptotic proteins.

**Fig 1:**
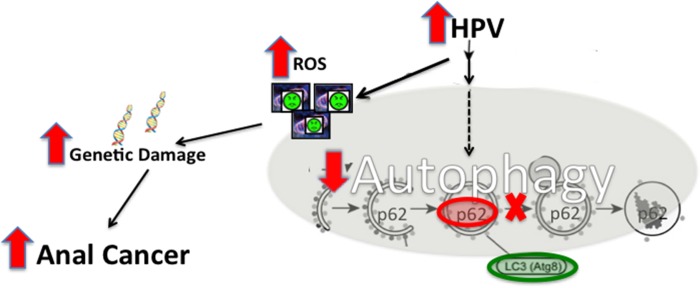
Representative diagram of proposed role of autophagy in anal carcinogenesis. This figure demonstrates inhibition of autophagy via HPV. With normal autophagic function p62 levels do not accumulate as it is continuously being degraded via the autophagic pathway. With late autophagic inhibition, there is blockage of the fusion of the autophagosome with the lysosome. This results in the accumulation of punctate LC3β and p62. There is also an accumulation of damaged organelles such as mitochondria which result in subsequent cellular and genetic damage and creates an environment that promotes carcinogenesis.

There is an abundance of evidence that several of the HPV-encoded oncoproteins modulate the autophagic response. E6 expression results in sustained protein kinase B (AKT)/mammalian target of rapamycin (mTOR) activity which can inhibit the autophagic pathway [5–7]. Both E6 and E7 activate the extracellular signal-related kinase (ERK) pathway, which has been shown to down regulate autophagy [8]. Finally, E7 inhibits the Jun amino-terminal kinase (JNK) pathway which also inhibits autophagy [9]. Taken together these inhibitory signals have profound effects on autophagy-mediated removal of intracellular pathogens and dysfunctional organelles, the protective function against genomic damage, and therefore result in changes that promote cancer development. These inhibitory actions by E6 and E7 on autophagy are adaptive to prevent viral clearance and the maintenance of cell viability. These properties imply an important role of autophagy in anal cancer *development* (carcinogenesis) as has been demonstrated in other HPV-associated cancers such as cervical cancer [10] (Fig 1). On the other hand, autophagy has been demonstrated to be upregulated in established tumors of various types [11] as a tumor survival mechanism to, for example, meet the high metabolic needs of the tumors cells. Therefore, there appears to be a tipping point where autophagy goes from being important for tumor prevention, to becoming important for tumor cell survival [12]. The goal of this study is to evaluate the autophagic response throughout anal carcinogenesis using two commonly examined autophagic proteins (LC3β and p62) as biomarkers for the autophagic process, along with electron microscopy to confirm the presence of autophagosomes and autophagolysosomes in order to correlate the state of autophagic flux (induction to degradation) with neoplastic progression [13]. In this study, we test the hypothesis that autophagy is inhibited in the anal epithelium resulting in carcinogenesis.

## Methods

### Mice

K14E6 and K14E7 mice have been previously described [14,15]. These mice express HPV-16 E6 or E7 oncoprotein in their epithelium, respectively. These two strains of mice were bred to each other to generate mice carrying both transgenes in the heterozygous state (heretofore called K14E6/E7 mice). All mice used in this study were maintained on the inbred FVB/N genetic background. Mice were maintained in an IACUC approved facility with 12 hour light/dark cycle, ad libitum food and water as well as HEPA filtered air.

### 7,12 dimethylbenz[a]anthracene (DMBA) induced anal carcinogenesis in K14E6/E7 and FVB/N mice

Weekly topical application of 0.12μmole of DMBA (60% acetone/40% dimethylsulfoxide (DMSO)), starting at 5 weeks of age, to the anus of double transgenic mice (K14E6/E7) and nontransgenic (FVB/N) mice was performed as previously published [3]. Both genotypes were treated for several weeks (5, 10, 15, or 20 weeks) with 25 mice per time point per genotype. Age matched control FVB/N and K14E6/E7 mice not treated with DMBA at identical time points (5, 10, 15 or 20 weeks) with 25 mice per group per time period per genotype were also studied.

Although not typical, we anticipated that tumors that develop in our animals could potentially cause discomfort to the animal. Animals do not receive routine administration of analgesia as discomfort is not common in this tumor phenotype. Our primary course of action when we see signs of animal distress (animal is scratching, licking, or biting tumor site, or shows pain response to palpation of the tumor) is humane euthanasia. The strain placed on the animal for feeding and drinking was decreased by placing seeds and/or moistened regular chow directly in the cage and providing water in bottles in addition to the automated watering system. Most mice were removed from the study at a pre-determined time point, prior to developing any untoward tumor related morbidity. Any mouse that became lethargic, unresponsive or extremely underweight, defined as more than 15% weight loss, was removed from the study. Mice with body scores <2 required approval and supervision from a Research Animal Resource Center (RARC) veterinarian for continued husbandry if experimental needs warranted delaying euthanasia. [14]. Due to the underlying genetic modifications, some of our mice developed a large thymus, which causes them to breathe more deeply than other strains of mice. Therefore, this is not a criterion for removal in our mice containing the E7 oncoprotein. However, if extreme dyspnea causing distress was identified the mouse was euthanized. In addition, as per the Medical School Animal Care and Use Committee (ACUC) policy on tumor burden, mice were euthanized under the following conditions: (1) A tumor causing impediment to the movement or bodily functions of the animal, (2) A tumor causing the animal to lose more than 15% of body weight, or loss of body condition resulting in body score of <2, or (3) Animal exhibits extreme lethargy such that they are unresponsive to mild stimulus.

In accordance with Public Health Service (PHS) policy for humane euthanasia, mice must be placed in a non-precharged chamber and 100% CO2 must be introduced at a rate of 10–30% of the chamber volume per minute as regulated by a flow meter attached to the CO2 canister. Death will be confirmed by cardiac and respiratory arrest. In adult rodents, respiratory arrest has been observed for several minutes; the eyes have lost color and are no longer wet. In animals less than 20 days old, respiratory arrest has been observed for 30 minutes or mice were decapitated. If tissue collection is required immediately, cervical dislocation is done after respiratory arrest has been observed to ensure death.

The number of animals needed per group were determined based on power analysis to detect at least a two-fold difference, with an alpha <0.05 and beta error of 80% between the treated and untreated groups, resulting in 25 mice per group.

### Histological Analysis of mouse anal tissues

All mouse anal tissues were collected and fixed in 4% paraformaldehyde for 24 hours and then placed in 70% ethanol. After fixation, the tissues were processed, embedded in paraffin, and serially sectioned at 5μm thickness. Every 7^th^ section was stained with hematoxylin and eosin (H&E) and evaluated by a gastrointestinal fellowship trained, board-certified surgical pathologist for evidence of papillomas, dysplasia (low-grade versus high-grade), or invasive carcinoma (Graded 1–3; Grade 1 representing well-differentiated and Grade 3 designating poorly-differentiated carcinomas). All mouse samples, K14E6/E7 and FVB/N, underwent H&E staining and histological evaluation, except for the samples utilized for electron microscopy (not included in the 25 mice per group totals).

### Immunofluorescence for autophagic proteins LC3β and p62

Paraffin embedded sections of human and mouse tissues (K14E6/E7 and FVB/N) were deparaffinized, rehydrated, subjected to antigen retrieval with 10mM sodium citrate buffer pH 6.0 and heat, permeabilized with 0.2% Triton X-100, and blocked with 5% milk/5% donkey serum in PBS. To examine autophagy, the sections were stained with monoclonal rabbit antibody against LC3β, which detects both LC3β-1 and LC3β-II (Santa Cruz Dallas, TX; 1:50 in 5% milk/5% donkey serum in PBS) or monoclonal mouse antibody for p62 (Abcam Cambridge, MA; 1:200 in 5% milk/5% donkey serum in PBS) overnight at 4° Celsius. Sections were then washed and stained with donkey anti-rabbit Fluor 488 and donkey anti-mouse Fluor 594 (Life technologies Carlsbad, CA; 1:500 in 5% milk/5% donkey serum in PBS) for one hour at room temperature in the dark. Slides were then counter stained with DAPI. Slides were imaged using the Ziess Axio Imager M2 imaging system. Images at 10x, 20x, 40 x and 63x magnification were obtained for each sample. Each 20x merged image was imported into ImageJ version 2.0.0 (Fiji distribution) and underwent additional processing. Images were split in the three channels (488, 594 and DAPI). All images were thresholded using the default with dark background. The areas of interest were manually selected and then RawIntDen measures for the region of interest to measure the intensity of the fluorescent signal. The RawIntDent was then normalized for the area of the region selected (RawIntDen/Area).

### Electron microscopy of K14E6/E7 anal tissue

Two K14E6/E7 mice per treatment time point underwent analysis via electron microscopy. Given the need for different tissue processing these mice are not included in the 25 mice/time point for the power analysis and did not undergo analysis by immunofluorescence or H&E. Mouse anal sections were fixed in 2.5% glutaraldehyde, 2.0% paraformaldehyde buffered in 0.1 M sodium phosphate buffer at 4°C overnight. The fixed samples were rinsed 5 times in phosphate buffered saline (PBS), and post-fixed in 1% osmium tetroxide in 0.1M PBS overnight at room temperature, and rinsed in PBS as before. Dehydration was performed in a graded ethanol series (35, 50, 70, 80, 90% for 15 minutes each step, 95% for 30 minutes, 100% for 3 x 15 minutes) at room temperature and then transitioned in propylene oxide (PO) 2 x 7 minutes at room temperature. Fully dehydrated samples were infiltrated in increasing concentrations of PolyBed 812 and PO mixtures. Embedding and polymerization took place in fresh PolyBed 812 for 24 hours and Semi-thin sections (1 mm) were first stained with Richardson’s stain (methylene blue/Azure II) for light microscopic evaluation. The samples were then sectioned on a Leica EM UC6 ultramicrotome at 100nm. The sections were collected on copper, pioloform/carbon coated 2x1 slot grids, and post-stained in uranyl acetate and lead citrate. The sectioned samples were viewed at 80kV on a Philips CM120 transmission electron microscope, equipped with MegaView III camera (Olympus Soft Imaging System Lakewood, CO). Images were reviewed by a pathologist skilled in electron microscopy to confirm the presence or absence of autophagosomes and autophagolysosomes.

### Pharmacologic inhibition of autophagy in FVB/N mice

Chloroquine phosphate (Sigma, St. Louis, MO) was diluted in phosphate buffered solution (PBS) and filtered prior to administration. Twenty FVB/N mice were given 3.5 mg/kg dose of chloroquine via intraperitoneal injection (IP) five days of the week (Monday-Friday) for 20 weeks. 10 of these mice were treated with DMBA and the remaining 10 were not treated with DMBA. Pharmacologic controls were FVB/N mice that did not receive chloroquine, 10 were treated with DMBA and 10 were not treated with DMBA.

### Human samples

Human anal samples from our surgical pathology archives were obtained and histologically graded by a fellowship trained, gastrointestinal pathologist as either normal, low-grade dysplasia, or high-grade dysplasia. Ten samples per histological classification were subjected to immunofluorescence for autophagic proteins LC3β and p62 as described below.

### Statistical Analysis

Using IBM SPSS Statistics Version 22, statistical differences in histological classification between mouse groups by genotype and in p62 and LC3β immunofluorescent intensity from Image J/FIJI was determine by one-way ANOVA. Statistical significance was defined as a p-value ≤ 0.05.

### Study Approval

All mice were maintained in an American Associated for Accreditation of Laboratory Animal Care-approved Wisconsin Institute for Medical Research (WIMR) Animal Care Facility. This study was carried out in strict accordance with the recommendations in the Guide for the Care and Use of Laboratory Animals of the National Institutes of Health and under the guidelines of the animal protocol that was approved by the School of Medicine and Public Health Institutional Animal Care and Use Committee at the University of Wisconsin-Madison (protocol number M02635- expiration 12/1/2017). De-identified human anal samples were obtained from the Department of Pathology and Laboratory Medicine. An IRB protocol was submitted and exemption obtained (Project Number 2015–0757) from the Health Sciences Institutional Review Board at the University of Wisconsin-Madison.

## Results

### K14E6/E7 mice, unlike non-HPV transgenic mice (FVB/N), develop DMBA induced anal cancer

**Fig 2:**
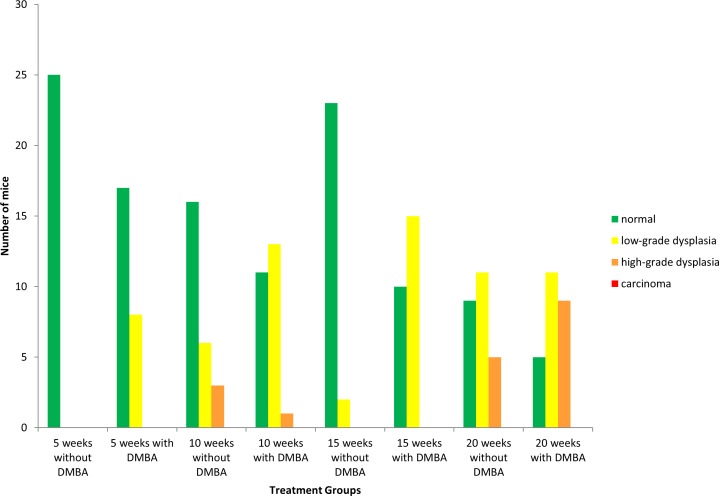
Histological analysis of the anal transition zone at various time points with and without DMBA in FVB/N mice. Anal histology identified by a trained pathologist for each time point (25 mice/group) following treatment with and without DMBA (0.12μmole topically to anus weekly). There is evidence of increased number of animals with high-grade dysplasia, however overt carcinoma is not seen with increasing treatment times with DMBA in mice without HPV oncogenes.

**Fig 3:**
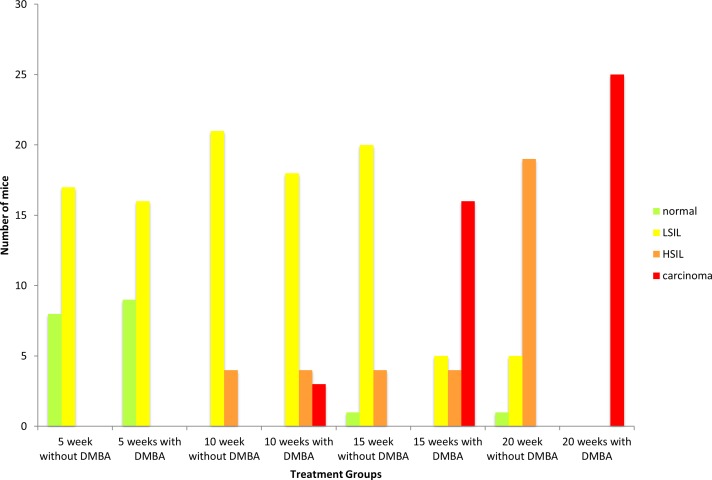
Histological analysis of the anal transition zone at various time points of DMBA treatment in K14E6/E7 mice. Anal histology identified by a trained pathologist for each time point (25 mice/group) following treatment with and without DMBA (0.12μmole topically to anus weekly). There is a statiscally significant increase in anal dysplasia over the time course of DMBA treatment, with the majority of the mice at 5 and 10 weeks of DMBA treatment having low-grade dysplasia, while 75% of mice at 15 weeks. By 20 weeks of DMBA treatment 100% of K14E6/E7 mice have overt carcinoma. During this same time course none of the K14E6/E7 mice not treated with DMBA developed anal cancer.

**Fig 4:**
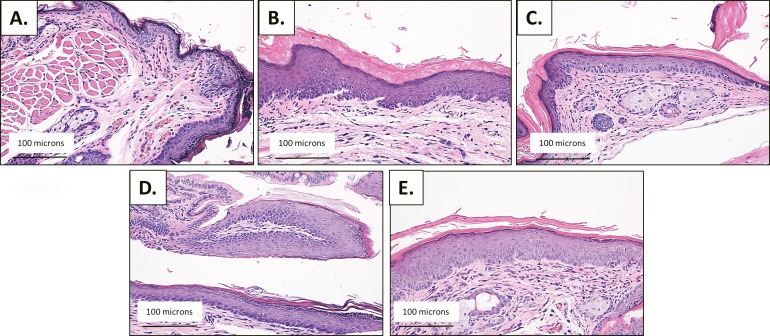
Histological analysis of the anal transition zone at various time points in FVB/N mice during DMBA treatment time course. Anal histology identified by a trained pathologist for each time point (25 mice/group) following treatment with and without DMBA (0.12μmole topically to anus weekly). A) H&E staining of animals not treated with DMBA at the 5 week timepoint reveals normal epithelium. (B) Following 5 weeks of DMBA treatment, there is inflammation seen on H&E staining. (C) At 10 weeks of DMBA treatment there is histological evidence of low-grade dysplasia. (D) Low-grade dysplasia is present at 15 weeks of DMBA treatment and (E) high-grade dysplasia at 20 weeks of DMBA treatment. All images are acquired at 20x magnification.

**Fig 5:**
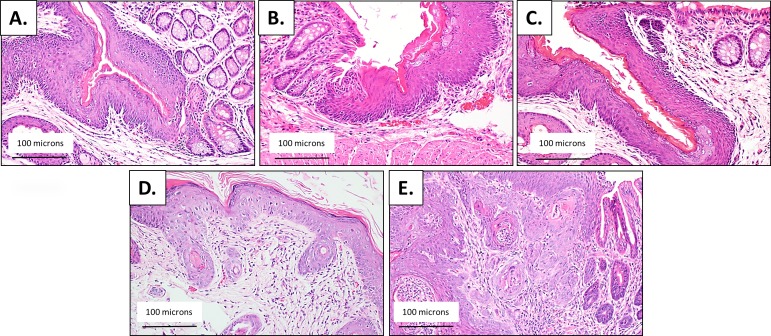
Histological examination of anal transition zone (ATZ) of K14E6/E7 double transgenic mice throughout a time course of no treatment versus DMBA treatment. (A) H&E staining of animals not treated with DMBA at the 5 week timepoint reveals normal epithelium. (B) Following 5 weeks of DMBA treatment, there is inflammation on H&E staining. (C) At 10 weeks of DMBA treatment there is histological evidence of low-grade dysplasia. (D) High-grade dysplasia is present at 15 weeks of DMBA treatment and (E) invasive squamous cell carcinoma at 20 weeks of DMBA treatment. All images are acquired at 20x magnification.

To monitor the role of autophagy in HPV-associated anal carcinogenesis we made use of HPV16 double transgenic mice (K14E6/E7) and background controls (FVB/N) at four time points (5, 10, 15, and 20 weeks). The distribution of histological classifications (normal, low-grade dysplasia, high-grade dysplasia or carcinoma) for each time point in both the DMBA treated and no DMBA FVB/N and K14E6/E7 mice is graphically depicted in Fig 2 and Fig 3, respectively. Representative H&E-stained tissue sections from the anal transitional zone of FVB/N and K14E6/E7 mice for each time point are shown in Fig 4 and Fig 5, respectively. Anal histology following treatment with DMBA resulted an increase in the number of cases with high-grade dyplasia which correlated with duration of weekly DMBA treatment for both genotypes (FVB/N and K14E6/E7). Again the FVB/N background controls do not develop overt carcinoma, which is supported by our studies. At 15 weeks of DMBA treatment, greater than 75% of K14E6/E7 animals had histological evidence of invasive carcinoma. By 20 weeks of DMBA treatment, 100% of the K14E6/E7 mice had developed invasive carcinoma. ANOVA analysis demonstrated a significant difference in the histological classifications between the K14E6/E7 DMBA treatment groups (p<0.01) as well as between the K14E6/E7 DMBA treatment groups and their associated no DMBA treatment controls (p<0.01). None of the FVB/N mice (number = 200) developed anal cancer over the twenty weeks of DMBA treatment which is consistent with prior studies and demonstrates the importance of the HPV16 oncogenes (E6 and E7) providing the requisite environment for the develop of anal cancer [3].

### Evidence for autophagic dysfunction with the development of low-grade dysplasia, but not in later stages of disease (high-grade dysplasia and caricnoma) for both human and K14E6/E7 mouse anal samples

Autophagy is a multi-step process that involves the formation of specialized orgenelles, autophagosomes, followed by formation of autophagolysosome creation (fusion of autophagsome with lysosome), a process refered to as autophagic flux. Autophagic flux can be followed by the use of two autophagic biomarkers, LC3β and p62. LC3β is a cellular protein that upon autophagic induction becomes incorporated into the autophagosome membrane, resulting in punctate appearance upon visualizaiton by immunoflourescence. p62 is a ubiquitin-binding protein which is degraded specifically via the autophagic pathway, and therfore will accumulate with autophagic dysfunction and decrease in its levels when the autophagic pathway is induced [13]. Another method for monitoring autophagy is western blot analysis assessing the conversion of LC3β-I to LC3β-II. Unfortunately, due to the miniscule size of the anorectal transition zone of the mouse anal specimen and the limited tissue from human archived samples, this methodology could not be utilized. To overcome this limitation electron microscopy was performed to monitor for the presence of autophagosomes and autophagolysosomes in the double transgenic mice.

**Fig 6:**
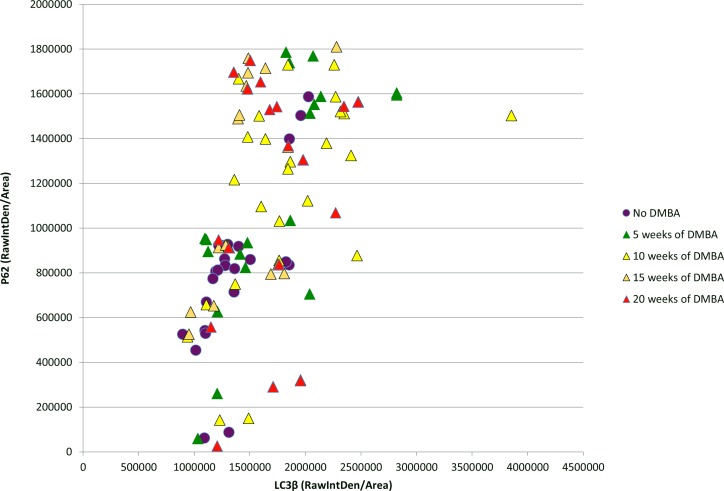
LC3β and p62 immunofluorescence levels for each FVB/N mouse over the DMBA treatment time course as determined by FIJI analysis of 20x images. The above graph shows that in general FVB/N treatment groups are very similar to each other without profound differences in autophagic function. ANOVA analysis demonstrated a statistically significant increase in only in LC3β at 10 weeks of DMBA treatment compared to no DMBA treated mice (p-value = 0.001), indicating increase in autophagic function at this one time point compared to others. There were no significant differences with regards to p62 levels.

Dual immunoflourescent staining and high magnification imaging (20x and 40x) was performed to identify autophagic induction with the formation of punctate LC3β and autophagic function with corresponding p62 levels. We began by examining the nontransgenic FVB/N samples with FIJI software. Each mouse is plotted in Fig 6 with LC3β immunoflourescent intensity on the x-axis and p62 immunoflourescent intensity on the y-axis. ANOVA analysis demonstrated no signficant differences in p62 over this time course. There was a statiscally significant increase in LC3β at 10 weeks of DMBA compared to no DMBA treatment controls (p-value = 0.001). This indicates no evidence of autophagic dysfunction in the FVB/N mice with/without DMBA treatments.

**Fig 7:**
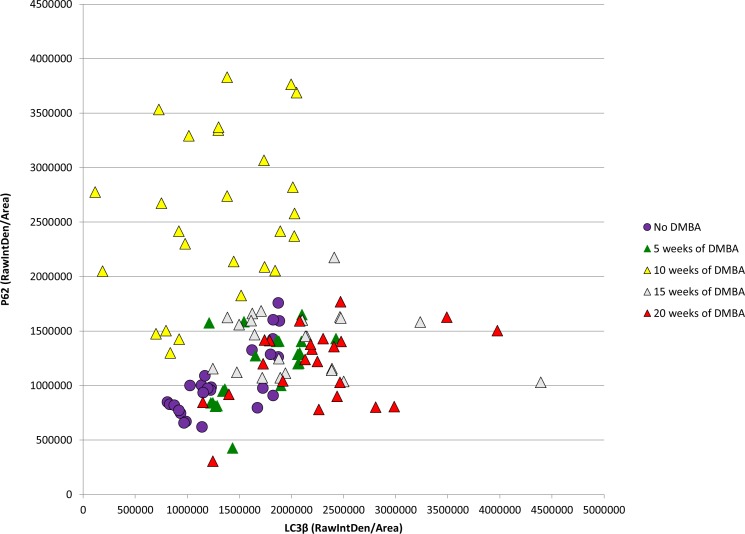
LC3β and p62 immunofluorescence levels in K14E6/E7 mice over DMBA treatment time course as determined by FIJI analysis. ANOVA analysis demonstrated a statistically significant increase in p62 levels at 10 weeks of DMBA treatment (yellow triangles) compared to ***all*** other treatment groups, indicating a blockage of autophagic degradation function. There was also a statistically significant increase in LC3β in the 15 and 20 week DMBA treated mice compared to no DMBA treated mice, demonstrating a significant increase in autophagic induction at these two time points. 10 weeks of DMBA treatment also showed an increase in LC3β levels compared to no treatment controls, but it did not reach statistical significance. The above graph shows that mice at the 10 week DMBA treatment time point (yellow triangles) are very different from the other treatment groups in terms of evidence of autophagic dysfunction.

**Fig 8:**
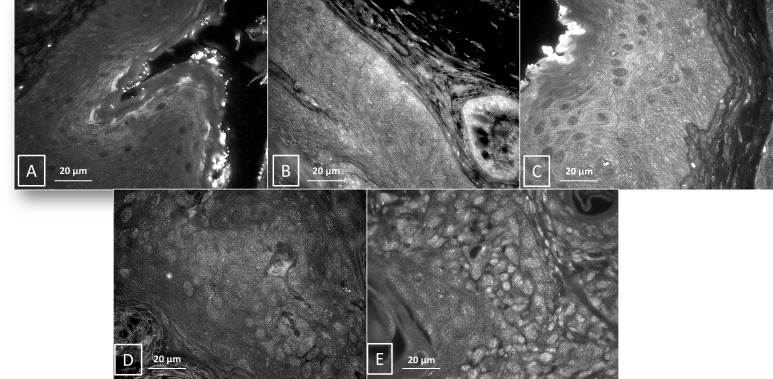
LC3β immunofluorescence staining of K14E6/E7 mice over the DMBA treatment time course with evidence of increased punctate LC3β noted at all treatment time points. (A). No DMBA treated K14E6/E7 mouse with low levels of autophagy can be seen by low levels of punctate LC3β expression. (B) With 5 weeks of DMBA treatment where again low levels of autophagy can be seen by low levels of punctate LC3β, (C) 10 weeks of DMBA treatment with increasing levels of punctate LC3β, (D) 15 weeks of DMBA treatment with continued increase in punctate LC3β, and (E) and 20 weeks of DMBA treatment there is also a continued increase in the extent of punctate of LC3β expression, indicating autophagic induction is intact in K14E6/E7 mice treated with DMBA.

**Fig 9:**
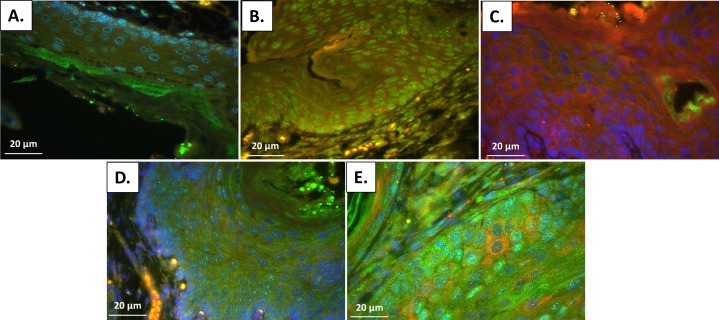
Immunofluorescence for autophagic proteins following DMBA treatment. Immunofluorescence for LC3β (cytoplasmic, green), p62 (cytoplasmic, red) and DAPI (nuclear, blue) at various time points of treatment. (A) At 0 weeks of treatment, there is a low level of punctate LC3β expression and minimal p62 noted, indicating low levels of autophagy in this untreated specimen. (B) After 5 weeks of DMBA treatment, there is evidence of autophagic induction with the increase in punctate, granular LC3β compared to 0 week treatment mice (A). (C) An overlay image with co-expression (LC3β and p62) expressed as orange at the 5 week time point compared to LC3β alone in panel B indicates a mild increase in p62 in addition to LC3β at this time point. (D) With 10 weeks of DMBA treatment, there is significant accumulation of p62 expression and an increase in punctate LC3β indicating autophagic induction without degradation of autophagy-specific substrate p62. (E) At 15 weeks of DMBA treatment, the time of development of high-grade dysplasia, p62 levels begin to decrease with continued evidence of punctate LC3β indicating autophagic induction with the ability to degrade p62. (F) At 20 weeks of treatment, where carcinoma is present, there is again a significant increase in LC3β punctate expression with low levels of p62. All images are acquired at 20x magnification.

A similar graphic comparison of K14E6/E7 mice at multiple time points was also performed and is shown in Fig 7. ANOVA analysis demonstrated a significant increase in p62 at 10 weeks of DMBA treatment compared to all other groups and 15 weeks of DMBA treatment compared to no DMBA treatment controls. There was a statiscally signficant increase in LC3β in the 15 and 20 week DMBA treatment groups over no DMBA treatment controls, with 10 weeks of DMBA treatment approaching statisical signficance with a p-value of 0.06. Figs 8 and 9 contain representative IF images of the K14E6/E7 treatment time course with Fig 8 staining only for LC3β to better demonstrate punctate LC3β and Fig 9 containing the same representative sample with co-staining for LC3β (green) and p62 (red) with DAPI nuclear stain (blue). Fig 8 panel A demonstrates low levels of punctate LC3β in no DMBA treated K14E6/E7 mice. Panel B shows that with 5 weeks of DMBA treatment evidence of increased levels of punctate LC3β that remains elevated at the 10 week DMBA time point (panel C), 15 week DMBA time point (panel D), and 20 week DMBA time point (panel E). Fig 9 shows the increase in p62 protein levels at 5 (panel B) and 10 weeks (panel C) compared to no DMBA controls (panel A), 15 weeks of DMBA treatment (panel D) and 20 weeks of DMBA treatment (panel E). These results indicate late autophagic dysfunction, with increased p62 levels in the setting of autophagosome formation, at 5 and 10 weeks of DMBA. At these two time points the majority of the mouse anal samples have low-grade dysplasia (Fig 3). At 15 and 20 weeks of DMBA treatment the majority of the anal samples show evidence of high-grade dysplasia or overt carcinoma (Fig 3) and at these later stages of anal carcinogenesis there are increased levels of punctate LC3β without the accumlation of p62. The increase in punctate LC3β without the accumlation of p62 indicates normal autophagic function. Considering the continuum of carcinogenesis from no disease to carconinoma in situ to overt carcinoma, analysis of the above indicates autophagic dysfunction in the early phases of carcinogenesis that perhaps resolves with higher grades of dysplasia and overt carcinoma in mice expressing HPV-encoded oncoproteins.

**Fig 10:**
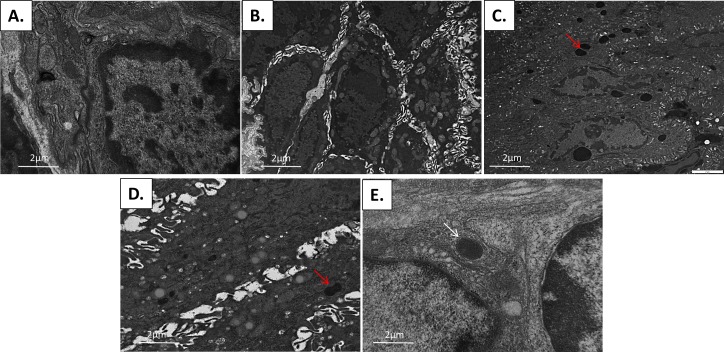
Electron microscopy following DMBA treatment. (A) Control K14E6/E7 animals with various organelles in normal quantities noted within the cell. (B) Following 5 weeks of DMBA treatment, there is an accumulation of mitochondria in the cell cytoplasm. (C) At 10 weeks, an accumulation of lysosomes is noted and is depicted with the red arrow. By 15 (D) and 20 (E) weeks of DMBA the accumulation of lysosomes is no longer present and there is evidence of autophagolysosomes (white arrow), which is formed after the autophagosome has fused with the lysosome.

Electron microscopy was performed on two K14E6/E7 anal samples per DMBA timepoint (0, 5, 10, 15, and 20 weeks of DMBA treatment) to monitor for the presence of autophagosomes and autophagolysosomes (Fig 10). K14E6/E7 mice not treated with DMBA (Fig 10A) were utlized as controls for comparison. Increased number of autophagosomes were observed within the epithelial cells following 5 weeks of treatment with DMBA (Fig 10B) without evidence of autophagolyosomes. At 5 weeks of DMBA treatment there were also noted increased numbers of mitochondria within autophagosomes. At 10 weeks of treatment with DMBA, an increase in the number of cellular lysosomes was noted (Fig 10C) in addition to the number of autophagosomes, but again, no autophagolyosomes were noted. This observation is consistent with an induction of autophagy with the inability of the autophagosome to fuse with the lysosome (later stage in autophagic pathway). By week 15 and 20 weeks of treatment with DMBA (Fig 10D and 10E), the number of lysosomes normalized and autophagolysosomes are seen (white arrow in Fig 10E), suggesting that autophagy is able to proceed on to completion. These results are consistent with the LC3β and p62 protein expression findings seen on immunofluorescent analysis of the K14E6/E7 mouse DMBA time course where initial autophagic dysfunction recovers with pathological advancement of disease (Figs 7–9).

**Fig 11:**
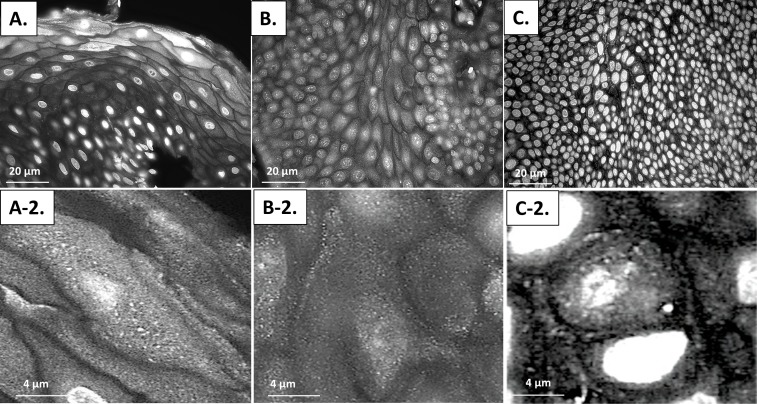
LC3β immunofluorescence staining of human samples to identify autophagic induction at various histological stages of carcinogenesis. Panels A-C are at 40x magnfication while Panels A-2 to C-2 are acquired at 63x magnification. Panel A contains a representative normal human anal specimen with evidence of punctate LC3β. Panel B demonstrates continued LC3β punctate formation with low-grade squamous intraepithelial lesion (LSIL) which equates to low-grade dysplasia in the mouse samples. Panel C depicts a human anal sample with high-grade squamous intraepithelial lesion (HSIL) which equates to high-grade dysplasia in the mouse samples, and continues to show evidence of punctate LC3β. These images demonstrate, as in the K14E6/E7 mouse samples, that autophagic induction is intact throughout anal carcinogenesis.

**Fig 12:**
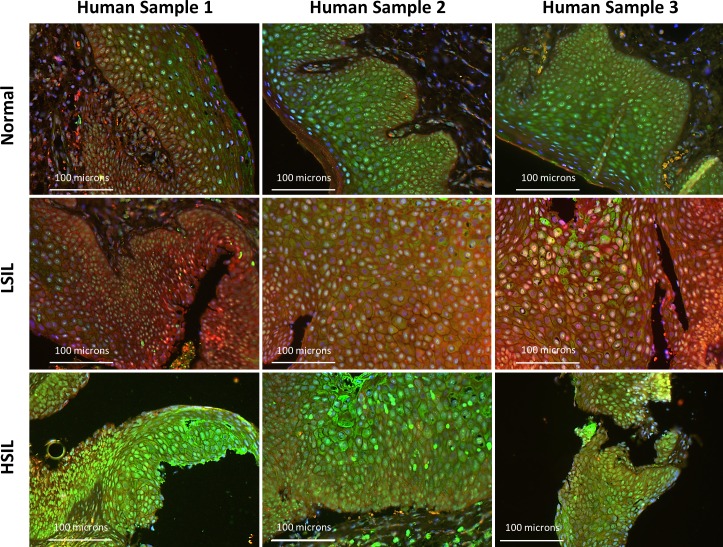
Immunofluorescence for autophagic proteins for human anal samples. Immunofluorescence for LC3β (cytoplasmic, green), p62 (cytoplasmic, red) and DAPI (nuclear, blue) for human anal samples with evidence of normal, LSIL, or HSIL. There are three distinct samples per histological subtype represented in this figure. There is low expression of LC3β and no to low expression of p62 in the normal anal samples. With the development of LSIL, there is significant accumulation of p62 expression in addition to punctate LC3β expression. In cases of of HSIL, there is again a significant increase in punctate LC3β protein expression, but with normalization of p62 levels. All images are acquired at 20x magnification. These images show evidence of autophagic dysfunction, similar to that seen in K14E6/E7 mouse anal samples.

**Fig 13:**
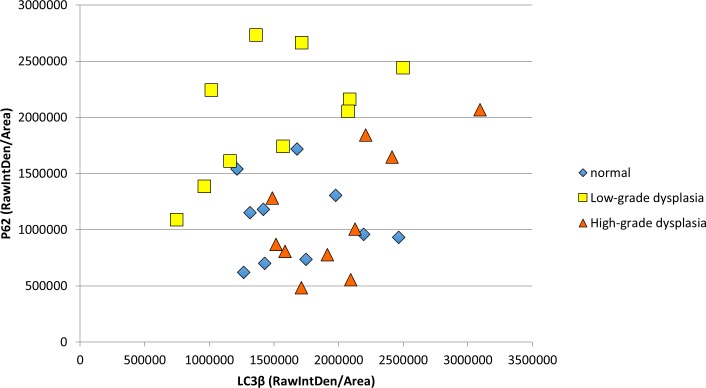
LC3β and p62 immunofluorescence intensity of human anal samples based on histological classification demonstrating autophagic dysfunction, similar to K14E6/E7 mice, with the development of low-grade dysplasia that is not evident in normal or high-grade dysplasia samples. There is statistical difference in p62 expression levels between normal and LSIL and between HSIL and LSIL (p-value = 0.001). The expression levels of LC3β and p62 show similar changes as noted in K14E6/E7 mice over the time course of DMBA treatment (Fig 7) with evidence of autophagic dysfunction in samples with low-grade dysplasia.

To determine if a similar pattern of autophagic dysfunction is present in human samples throughout carcinogenesis, we examined levels of punctate LC3β and p62 in 30 human specimens with normal histology through high-grade squamous intraepitheal lesion (HSIL) (10 samples/histological classification) (Figs 11 and 12). Fig 11 demonstrates immunoflourscent images stained only for LC3β to determine if punctate LC3β can be visualized. The upper panels (A-C) are at 40x magnfication while the lower panels (A2-C2) are 63x magnification of the same samples in the upper panel. Panel A demonstrates low levels of punctate LC3β in normal human anal tissue samples. Panel B demonstrates an increase in punctate LC3β in samples with low-grade squamous intraepthelial lesion (LSIL), which equates to low-grade dysplasia histology for the mouse samples. Panel C demonstrates evidence of continued punctate LC3β in samples with high-grade squamous intraepithelial lesion (HSIL), which equates to high-grade dysplasia in the mouse samples. Fig 12 contains three representative images from each histological classification that have undergone dual staining for LC3β (green) and p62 (red). The normal anal mucosa demonstrates baseline staining for LC3β including punctate staining of LC3β with low levels of p62, indicative of low levels of autophagy. In LSIL, there was a further increase in overall LC3β levels and punctate staining of LC3β, but also a much larger increase in levels of p62 that is seen in all samples indicating a block in autophagic degradation. Similar to the mice, autophagic degradative capability was restored in HSIL as evidenced by increases in LC3β protein expression compared to the normal tissue and a normalization of p62 levels to a normal tissue equivalent. These images were also analyzed utilizing Image J/FIJI. Fig 13 shows the immunoflourescent intensity as measured by FIJI for both LC3β on the x-axis and p62 on the y-axis. ANOVA analysis was performed. There were statistically signficant differences in p62 levels between normal and LSIL (p-value = 0.006). There were statiscally signficant differences in p62 levels between normal and LSIL and between HSIL and LSIL (p-value = 0.001) indicating autophagic dysfunction with low-grade dysplasia that is not present in normal or high-grade dysplasia samples. There was no statistically signficant difference in p62 levels between normal and HSIL (p-value = 0.973). In terms of LC3β expression, there was no statistically signficant difference between any of the groups (p-value > 0.05). There was insufficient tissue from archived samples to perform electron microscopy or western blot analysis. These finding are consistent with the K14E6/E7 mouse data.

### Pharmacologic autophagic inhibition in FVB/N results in DMBA induced carcinogenesis similar to K14E6/E7 DMBA induced carcinogenesis

**Fig 14:**
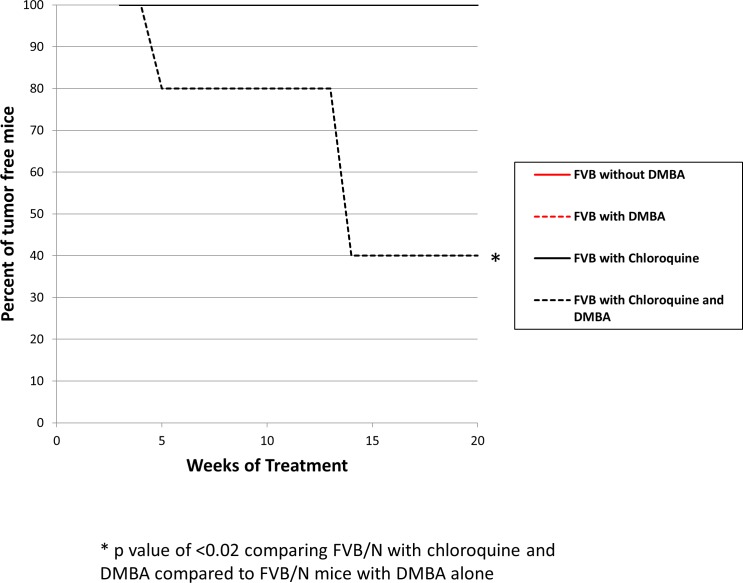
Percent Tumor Free Survival curves for FVB/N mice with and without DMBA and chloroquine treatment. The curves in black represent FVB/N mice with and without DMBA that did not receive chloroquine. The curves in red are FVB/N mice receiving chloroquine with and without DMBA. There is a statistically significant increase in the number of FVB/N mice that developed anal tumors with DMBA treatment when the mice were also treated with the late autophagic inhibitor, chloroquine. Both FVB/N mice without DMBA and with chloroquine alone did not develop anal tumors over the time course, resulting in the lines overlapping.

**Fig 15:**
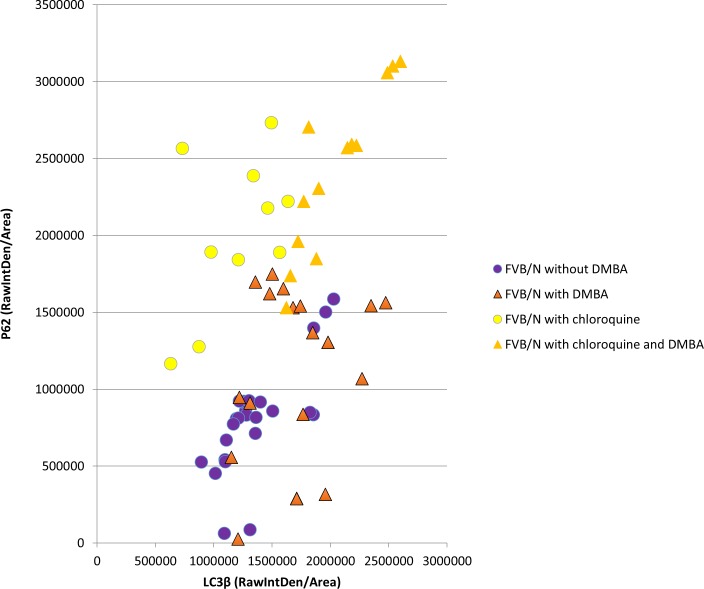
LC3β and p62 immunofluorescence intensity in FVB/N mice treated with and without DMBA and with and without a late autophagic inhibitor demonstrating the importance of autophagy in anal carcinogenesis. FVB/N mice treated with DMBA alone do not develop anal cancer (red triangles). However, with autophagic inhibition 100% of FVB/N mice developed anal tumors with DMBA treatment (orange triangles). ANOVA analysis demonstrated as statistically significant increase in p62 levels in mice received chloroquine with and without DMBA (yellow circles and orange triangles respectively). There was also a statisically significant increase in LC3β in FVB/N mice treated with DMBA alone (red triangles) and mice treated with chloroquine and DMBA (orange triangles) compared to those not treated with DMBA with and without chloroquine (yellow and blue circles, respectively).

None of the FVB/N mice, with and without DMBA, developed anal cancers (n = 200) in the absence of chloroquine treatment and none of the FVB/N mice treated with chloroquine alone (no DMBA) developed anal cancers (n = 10). FVB/N mice treated with chloroquine and DMBA (n = 10) showed signs of tumor development as early as five weeks of DMBA treatment. The difference in anal tumor development between FVB/N mice treated with DMBA alone versus FVB/N mice treated with chloroquine and DMBA was statistically significant (p-value = 0.02). The tumor free survival of FVB/N mice with and without DMBA and with/without chloroquine is shown in Fig 14. Fig 15 depicts p62 (y-axis) and LC3β expression of each mouse in the study. This figure shows a similar LC3β and p62 effect as seen in the K14E6/E7 mice at 10 weeks of DMBA treatment. ANOVA analysis was performed. There was a statiscally signifcant increase in p62 levels in mice that received chloroquine with or without DMBA for 20 weeks. In terms of LC3β expression there was a statisically signficant increase noted in FVB/N mice treated with DMBA compared to those not treated with DMBA or chloroquine. There was also a statisically significant increase in LC3β expression in FVB/N mice treated with DMBA alone and those treated with chloroquine and DMBA compared to those not treated with DMBA with/without chloroquine. These findings support the hypothesis that autophagic dysfunction promotes anal cancer development in the setting of DMBA, as is seen in the double transgenic mice treated with DMBA.

## Discussion

LC3β is an autophagic protein that is required for the formation of the autophagosome double membrane and is physically attached to the membrane during autophagosome formation (LC3β-II). LC3β protein levels increase during autophagic induction with expression patterns change from diffuse cytosolic staining to a granular, punctate staining during autophagosome formation, making LC3β punctate formation a good marker of autophagic induction. Autophagic induction can also be measured by the formation of autophagosomes as seen by electron microscopy (EM). EM is the current gold standard for identification of autophagy with the identification of autophagosomes at various stages throughout the autophagic process. Autophagosomes are double membrane vesicles where fusion of the outer layer to the lysosome creates an autolysosome or autophagolysosome forms. The lysosome degrades the autophagosome contents along with its inner membrane. p62, on the other hand, is an autophagic-specific substrate where levels increase during autophagic inhibition and decrease with autophagic induction. Thus, p62 is a useful marker of autophagic degradation [16]. In our HPV transgenic mouse model investigations, autophagic induction *without* degradation of autophagosome contents was noted as early as 5 weeks after the start of DMBA treatment. At 5 weeks of DMBA treatment, in the presence of low-grade dysplasia, there appeared to be an inhibition of late autophagic flux as evidenced by autophagosome formation with lack of evidence of fusion to the lysosome (no autophagolysosomes) based upon our EM studies and the accumulation of p62 by immunofluorescence. At 10 weeks of treatment, there was an accumulation of lysosomes, mitochondria and autophagosomes on EM, without evidence of autophagolysosomes. These, findings indicate autophagic dysfunction early in anal carcinogenesis (low-grade dysplasia). When high-grade dysplasia develops at 15 weeks of DMBA or invasive squamous cell carcinoma at 20 weeks of DMBA treatment, EM demonstrated autophagolysosome formation while there was normalization of p62 levels by IF displayed normalization of p62 levels. These findings indicate normalization of autophagic function later in anal carcinogenesis (high-grade dysplasia and carcinoma). These changes in autophagy which correlate with histological changes, displayed a similar pattern in our human anal samples.

To further investigate the role of autophagy in anal carcinogenesis, we utilized nontransgenic mice (FVB/N), which do not typically develop anal cancer with DMBA treatment, and used a pharmacologic inhibitor of autophagy (chloroquine). With the use of chloroquine in these mice, in the setting of DMBA treatments, all of the mice developed anal cancer over the 20 week time period similar to the HPV16 transgenic mice. These mice show similar LC3β and p62 accumulation as seen in the K14E6/E7 mice with low-grade dysplasia indicating autophagic dysfunction.

Autophagy plays an important role in maintaining cellular homeostasis by degrading damaged proteins and organelles that can result in cellular stress and damage. Under baseline conditions, autophagy occurs at a low level in all cells. With cellular stress (hypoxia, starvation, infection, etc.) autophagy is induced to in order restore the cellular homeostasis. For this response to be protective, it cannot only be induced, but is required to proceed to completion such that the autophagosomes fuses to the lysosome to form the autophagolysosome that then triggers degradation of its contents. From a viral standpoint, the inhibition of autophagy at any point of the autophagic process is an adaptive response that allows for viral survival and replication. Indeed, early on in infection, HPV has been shown to trigger a cellular autophagic response, which is thought to represent a host defense response to infection by these intracellular pathogens [17]. The ability of virally encoded oncogenes to inhibit the intact function of the autophagic pathway not only is likely to permit for infection to proceed, but also provides an environment that enhances cancer development.

In our HPV16 transgenic mice, the observations that in early neoplasia development there is induction in early stages of autophagy, while there is an inhibition of late stages of autophagy are consistent with the previous observations that HPV16 oncoproteins (E6 and E7) induce signals that are known to inhibit autophagy. Pharmacologic inhibition of autophagy can induce anal carcinogenesis in FVB/N mice that are not typically prone to tumor development even with carcinogen exposure. Future studies defining the specific activities of E6 and E7 that contribute to this inhibition of autophagy are necessary. Interestingly, our data show that in the later stages of neoplasia development, autophagic function normalizes. Our and other laboratory studies have shown that the maintenance of HPV-associated cancers is dependent upon the continued expression of the viral oncogenes [18–22] and in the context of our anal cancer model in these HPV16 transgenic mice, there is clear evidence for continued expression of the viral oncogenes in the cancers themselves [3]. Therefore, the release from inhibition of autophagy is unlikely to reflect a loss of expression of E6 and E7 during neoplasia progression; rather it must reflect other changes within the cancer cell that promote completion of the autophagic response. Autophagy has been implicated in helping make cancers resistant to traditional lines of chemotherapy [23]. Therefore, the goals of our future studies would be to identify the changes in late stages of neoplasia that allow for autophagy to proceed to completion and to determine whether inhibiting autophagy in anal cancers can improve therapeutic response.

## Supporting Information

S1 Table FVB with chloroquine tumor free survival. This table contains the raw data of FVB/N mice treated with and without DMBA and with and without chloroquine in terms of tumor free survival.
(XLSX)

S2 Table FVB FIJI LC3β p62 analysis with and without chloroquine. (XLSX)

S3 Table K14E6/E7 and FVB histology time course analysis. This table provides the histology for each mouse in the study based on genotype.
(XLSX)

S4 Table Human and K14E6/E7 FIJI LC3β p62 analysis. This table gives the raw data of LC3β and p62 levels measured by FIJI for each K14E6/E7 mouse and human sample in the study.
(XLSX)
